# ﻿Morpho-phylogenetic evidence reveals four novel species of *Coniella* (Diaporthales, Schizoparmaceae) from southern China

**DOI:** 10.3897/mycokeys.116.145857

**Published:** 2025-04-04

**Authors:** Duhua Li, Zixu Dong, Qiyun Liu, Yaling Wang, Zhaoxue Zhang, Xiuguo Zhang, Jiwen Xia

**Affiliations:** 1 College of Agriculture and Forestry, Linyi University, Linyi, Shandong, 276000, China Linyi University Linyi China; 2 College of Plant Protection, Shandong Agricultural University, Taian, Shandong, 271018, China Shandong Agricultural University Taian China

**Keywords:** Morphology, multigene phylogeny, new taxa, taxonomy

## Abstract

*Coniella* species are distributed worldwide and have been reported as plant pathogens, endophytes, or saprobes. In our ongoing survey of terrestrial plant fungi in southern China, we obtained *Coniella* isolates from diseased plant leaf tissues in Fujian, Hainan, and Yunnan provinces. Maximum likelihood and Bayesian inference based on four loci (ITS, LSU, *rpb2*, and *tef1-α*) were used to clarify the taxonomic placement of the species. We confirmed that they represent four new species, namely *Conielladiaoluoshanensis*, *C.dongshanlingensis*, *C.grossedentatae*, and *C.veri* based on both morphology and phylogeny support. The new species are compared with other *Coniella* species, comprehensive descriptions and micrographs are provided.

## ﻿Introduction

*Coniella* was formally introduced by Von Höhnel (1918) with *C.pulchella* (= *C.fragariae* (Oudem.) B. Sutton) as the type species (Von Höhnel 1918; Sutton 1977; Crous et al. 2014a). Samuels et al. (1993) initially recognized the uniqueness of *Schizoparme* and its relationship to *Coniella* and *Pilidiella*, these were initially placed in the Melanconidaceae. Both Castlebury et al. (2002) and Van Niekerk et al. (2004) revealed that these species within the Diaporthales, which they collectively designated as the *Schizoparme* complex. Rossman et al. (2007) introduced a new family, Schizoparmaceae, which comprises the distinctive teleomorph genus *Schizoparme*, its asexual state *Pilidiella*, and the closely related anamorph genus *Coniella*. These genera are cosmopolitan fungal pathogens associated with foliar, fruit, stem, and root diseases on a wide variety of hosts, including some economically important hosts (Van Niekerk et al. 2004; Alvarez et al. 2016). They occur as parasites on unrelated dicotyledonous hosts (Samuels et al. 1993) or sometimes as secondary invaders of injured plant tissues (Ferreira et al. 1997).

*Coniella* has undergone comprehensive morpho-molecular studies and experienced several taxonomic adjustments over the years. Petrak and Sydow (1927) classified *Coniella* into two subgenera: *Euconiella* (dark conidia), typified by *C.pulchella*, and *Pseudoconiella* (hyaline to pale conidia), typified by *C.granati*. Von Arx (1973, 1981) classified *Coniella* and *Pilidiella* as distinct genera, with *Coniella* characterized by dark brown conidia and *Pilidiella* by hyaline conidia that darken to a pale brown when mature. Nonetheless, Sutton (1980) and Nag Raj (1993) disregarded conidial pigmentation as a defining trait and still opted to employ the earlier name *Coniella*. Samuels et al. (1993) stated *Schizoparme* as the sexual morph and positioned it in Melanconidaceae. Castlebury et al. (2002) classified *Pilidiella* and *Coniella* as members of the *Schizoparme* complex. Van Niekerk et al. (2004) demonstrated that these taxa form a distinct evolutionary lineage within the Diaporthales based on ITS, LSU, and *tef1-α* sequences. Subsequently, Rossman et al. (2007) established a new family, Schizoparmaceae, including the above three genera, *viz. Coniella*, *Pilidiella*, and *Schizoparme*. Alvarez et al. (2016) demonstrated that *Coniella*, *Pilidiella*, and *Schizoparme* formed a monophyletic clade in Schizoparmaceae and suggested adopting *Coniella* (the older asexual typified name) instead of *Pilidiella* and *Schizoparme*, in accordance with Article 59.1 of the International Code of Nomenclature for Algae, Fungi, and Plants (ICN, Melbourne Code; McNeill et al. 2012). Additionally, due to the many numbers of species and the similarity in morphological characteristics, they suggested that the identification of new species within *Coniella* must be based on a combination of DNA sequence data and morphological characteristics. Chethana et al. (2017) used a combination of morphological analysis and multigene phylogeny with the genealogical concordance phylogenetic species recognition (GCPSR) method to delineate species boundaries. Hyde et al. (2020) and Tennakoon et al. (2021) conducted the recent phylogenetic analyses for *Coniella* species within the Schizoparmaceae. Currently, there are 66 accepted *Coniella* species (Index Fungorum: https://indexfungorum.org; MycoBank: http://www.mycobank.org; Mu et al. 2024).

In this study, we conducted extensive sample collection in southern China, primarily collecting plant leaves with obvious fungal necrosis or typical blight spot symptoms. Several *Coniella* fungi were collected from the diseased leaves of *Ampelopsisgrossedentata*, *Cinnamomumverum*, *Kadsuralongipedunculata*, and *Lygodiumcircinnatum*. Based on morphological and multi-locus analysis employing internal transcribed spacer (ITS), 28S large subunit ribosomal RNA gene (LSU), partial RNA polymerase II second largest subunit (*rpb2*), and translation elongation factor 1-alpha gene (*tef1-α*), four new *Coniella* species, namely *C.diaoluoshanensis*, *C.dongshanlingensis*, *C.grossedentatae*, and *C.veri*, were proposed.

## ﻿Materials and methods

### ﻿Sample collection and isolation

During 2022 to 2024, a large number of plant leaves that exhibited obvious signs of fungal necrosis or typical blight spot symptoms were collected from Fujian, Hainan, and Yunnan provinces in China. This study used tissue isolation methods to isolate fungi (Li et al. 2024). These diseased leaves were cut into small pieces of about 25 mm^2^ and surface sterilized by immersion in a 75% ethanol solution for 60 s, washed one time in sterile deionized water for 20 s, transferred to 5% sodium hypochlorite (NaOCl) for 90 s, and then washed three times in sterile deionized water for 60 s, subsequently dried on sterilized filter paper. The tissue pieces were transferred to the potato dextrose agar (PDA, 200 g potato, 20 g dextrose, 20 g agar, add deionized water and fill to 1000 mL, natural pH) plates and placed in a biological incubator at 25 °C for 3–4 days. The hyphal tips of individual colonies were transferred to new PDA plates to obtain pure cultures, which were then cut into 25 mm^2^ pieces using a sterile scalpel and stored in 2 mL frozen tubes containing 20% sterilized glycerin, with 8–10 pieces placed in each tube, for fungal strain preservation at -20 °C for further study.

### ﻿Morphological and cultural characterization

The culture characteristics of the colonies were observed and photographed using a Sony Alpha 6400L digital camera (Sony Group Corporation, Tokyo, Japan) on 7 and 14 days, respectively. The micromorphological characteristics of the colonies were observed with the Olympus SZX10 stereomicroscope and Olympus BX53 microscope (Olympus Corporation, Tokyo, Japan), along with the BioHD-A20c color digital camera (FluoCa Scientific, China, Shanghai). Structural measurements were carried out using Digimizer software (v5.6.0) with a minimum of 30 measurements taken for each structure, such as conidiophores, conidiogenous cells, and conidia. The voucher specimens have been deposited in the Herbarium of the Department of Plant Pathology, Shandong Agricultural University, Taian, China (HSAUP). Additionally, the ex-type living cultures were deposited in the Shandong Agricultural University Culture Collection (SAUCC) and the China General Microbiological Culture Collection Center (CGMCC). The taxonomic information of the new taxa were submitted to MycoBank (http://www.mycobank.org, accessed on 2 Jan. 2025).

### ﻿DNA extraction, PCR amplification, and sequencing

The DNA of the fungal genome was extracted using the modified cetyltrimethylammonium bromide (CTAB) method (Guo et al. 2000; Wang et al. 2023) or the magnetic bead kit method (OGPLF-400, GeneOnBio Corporation, Changchun, China) (Zhang et al. 2023). PCR amplifications of four genes (ITS, LSU, *rpb2*, and *tef1-α*) were done, and the corresponding primer pairs and PCR conditions were listed in Table 1. The PCR reaction was conducted in a 12 μL reaction volume, with a composition of 6 μL of 2 × Hieff Canace® Plus PCR Master Mix (with dye) (Cat. No. 10154ES03, Yeasen Biotechnology, Shanghai, China), 0.5 μL each of forward and reverse primer (10 μM TsingKe, Qingdao, China), and 0.5 μL of template genomic DNA (about 10 ng/μL), with the volume adjusted to 12 μL using distilled deionized water. PCR products were separated using 1% agarose gel and GelRed (TsingKe, Qingdao, China). Gel extraction was purified using a Gel Extraction Kit (Cat. No. AE0101-C, Shandong Sparkjade Biotechnology Co., Ltd., Jinan, China). The purified PCR products were subjected to bidirectional sequencing by Sangon Biotech Company Limited (Shanghai, China). The raw data were analyzed using MEGA v. 7.0 to obtain consistent sequences (Kumar et al. 2016). The sequence data have been deposited in GenBank, and their accession numbers were listed in Table 2.

**Table 1.** The primer sequences and PCR programs in this study.

Locus	Primers	Sequence (5’ – 3’)	PCR cycles	References
ITS	ITS5	GGA AGT AAA AGT CGT AAC AAG G	(94 °C: 30 s, 55 °C: 30 s, 72 °C: 45 s) × 29 cycles	White et al. 1990
	ITS4	TCC TCC GCT TAT TGA TAT GC		
LSU	LR0R	GTA CCC GCT GAA CTT AAG C	(94 °C: 30 s, 48 °C: 50 s, 72 °C: 1 min 30 s) × 35 cycles	Vilgalys and Hester 1990; Rehner and Samuels 1994
	LR5	TCC TGA GGG AAA CTT CG		
*rpb2*	RPB2-5F2	GGG GWG AYC AGA AGA AGG C	(94 °C: 45 s, 60 °C: 45 s, 72 °C: 2 min) × 5 cycles, (94 °C: 45 s, 54 °C: 45 s, 72 °C: 2 min) × 30 cycles	Liu et al. 1999; Sung et al. 2007
	RPB2-7CR	CCC ATR GCT TGY TTR CCC AT		
*tef1-α*	EF1-728F	CAT CGA GAA GTT CGA GAA GG	(95 °C: 30 s, 51 °C: 30 s, 72 °C: 1 min) × 35 cycles	O’Donnell et al. 1998; Carbone and Kohn 1999
	EF2	GGA RGT ACC AGT SAT CAT GTT		

**Table 2.** Species names, strain numbers, hosts or substrates, regions, and corresponding GenBank accession numbers of DNA sequences used in this study.

Species	Strain numbers	Host/Substrate	Region	GenBank accession numbers				References
				ITS	LSU	*rpb2*	*tef1-α*	
*Coniellaafricana*	CBS 114133* = CPC405	*Eucalyptusnitens*	South Africa	AY339344	AY339293	KX833421	KX833600	Van Niekerk et al. 2004; Alvarez et al. 2016
*Coniellacastanea*	SAUCC200313*	*Castaneamollissima*	China	OL757537	OL757563	OL770463	OL780610	Wang et al. 2022
	SAUCC200314	*Castaneamollissima*	China	OL757538	OL757564	OL770464	OL780611	Wang et al. 2022
*Coniellacili*	GUCC 194020.1	*Rosaroxburghii*	China	ON791171	ON791212	ON815908	ON815944	Zhang et al. 2024b
	GUCC 196007.1*	*Rosaroxburghii*	China	ON791172	ON791213	ON815909	ON815945	Zhang et al. 2024b
*Coniellacrousii*	NFCCI 2213	*Terminalia chebula*	India	HQ264189	NA	NA	NA	Rajeshkumar et al. 2011
***Conielladiaoluoshanensis***	CGMCC3.27786* = SAUCC 7481-1	***Kadsuralongipedunculata***	**China**	**PQ357094**	**PQ357134**	**PQ361030**	**PQ404804**	**This study**
	**SAUCC 7481-4**	***Kadsuralongipedunculata***	**China**	**PQ357095**	**PQ357135**	**PQ361031**	**PQ404805**	**This study**
*Conielladiospyri*	CBS 145071* = CPC 34674	*Diospyrosmespiliformis*	South Africa	MK047439	MK047489	MK047543	MK047562	Crous et al. 2018
*Conielladiplodiella*	CBS 111858* = CPC3708	*Vitisvinifera*	France	AY339323	KX833335	KX833423	KX833603	Van Niekerk et al. 2004; Alvarez et al. 2016
	CBS 112729 = CPC3927	*Vitisvinifera*	South Africa	KX833520	KX833345	KX833433	KX833613	Alvarez et al. 2016
*Conielladiplodiopsis*	CBS 109.23 = CPC 3933	*Vitisvinifera*	Switzerland	NA	AY339287	KX833440	KX833624	Van Niekerk et al. 2004; Alvarez et al. 2016
	CBS 590.84* = CPC 3940	*Vitisvinifera*	Italy	AY339334	AY339288	NA	NA	Van Niekerk et al. 2004
	CBS 116310 = CPC 3793	*Vitisvinifera*	Italy	KX833532	KX833357	KX833443	KX833627	Alvarez et al. 2016
***Conielladongshanlingensis***	CGMCC3.27785* = SAUCC 7265-5	***Lygodiumcircinnatum***	**China**	**PQ357090**	**PQ357130**	**PQ361026**	**PQ404800**	**This study**
	**SAUCC 7265-6**	***Lygodiumcircinnatum***	**China**	**PQ357091**	**PQ357131**	**PQ361027**	**PQ404801**	**This study**
*Conielladuckerae*	CBS 142045*= VPRI 13689	*Lepidospermumconcavum*	Australia	KY924929	NA	NA	NA	Marin-Felix et al. 2017
*Coniellaerumpens*	CBS 523.78*	Rotten wood	Chile	KX833535	KX833361	KX833446	KX833630	Alvarez et al. 2016
*Coniellaeucalyptigena*	CBS 139893* = CPC 24793	*Eucalyptusbrassiana*	Malaysia	KR476725	KR476760	NA	NA	Crous et al. 2015a
*Coniellaeucalyptorum*	CBS 112640* = CPC 3904 = DFR 100185	*Eucalyptusgrandis* × *E.tereticornis*	Australia	AY339338	AY339290	KX833452	KX833637	Van Niekerk et al. 2004; Alvarez et al. 2016
	CBS 114852	*Eucalyptus* sp.	Australia	KX833556	KX833380	KX833464	KX833652	Alvarez et al. 2016
*Coniellafici*	MFLU 18-2578*	*Ficusseptica*	China	MW114356	MW114417	NA	NA	Tennakoon et al. 2021
*Coniellafragariae*	CBS 172.49* = CPC 3930	*Fragaria* sp.	Belgium	AY339317	AY339282	KX833472	KX833663	Van Niekerk et al. 2004; Alvarez et al. 2016
	CBS 454.68	*Malussylvestris*	Denmark	KX833571	KX833393	KX833477	KX833670	Alvarez et al. 2016
*Coniellafujianensis*	CGMCC3.25353	*Canariumalbum*	China	OR623057	OR623054	OR637413	OR637415	Mu et al. 2024
	CGMCC3.25354*	*Canariumalbum*	China	OR623058	OR623055	OR637414	OR637416	Mu et al. 2024
*Coniellafusiformis*	CBS 141596* = CPC 19722	*Eucalyptus* sp.	Indonesia	KX833576	KX833397	KX833481	KX833674	Alvarez et al. 2016
	CBS 114850	*Eucalyptuspellita*	Australia	KX833574	KX833395	KX833479	KX833672	Alvarez et al. 2016
*Coniellagranati*	CBS 132860	*Punicagranatum*	Turkey	KX833577	KX833400	KX833484	KX833677	Alvarez et al. 2016
	CBS 252.38 = ATCC 12685 = CPC 3714	*Vitisvinifera*	Italy	KX833581	AY339291	KX833488	KX833681	Van Niekerk et al. 2004; Alvarez et al. 2016
***Coniellagrossedentatae***	**SAUCC 1354-1**	***Ampelopsisgrossedentata***	**China**	**PQ357062**	**PQ357102**	**PQ361000**	**PQ404774**	**This study**
	CGMCC3.27783*= SAUCC 1354-3	***Ampelopsisgrossedentata***	**China**	**PQ357063**	**PQ357103**	**PQ361001**	**PQ404775**	**This study**
*Coniellaheterospora*	CBS 143031* = FMR 15231	Herbivorous dung	Spain	LT800501	LT800500	LT800502	LT800503	Crous et al. 2017
*Coniellahibisci*	CBS 109757* = AR 3534	*Hibiscus* sp.	Africa	KX833589	AF408337	NA	KX833689	Castlebury et al. 2002; Marin-Felix et al. 2017
*Coniellajavanica*	CBS 455.68*	*Hibiscussabdariffai*	Indonesia	KX833583	KX833403	KX833489	KX833683	Alvarez et al. 2016
*Coniellakoreana*	CBS 143.97*	NA	South Korea	KX833584	AF408378	KX833490	KX833684	Alvarez et al. 2016
*Coniellalanneae*	CBS 141597* = CPC 22200	*Lannea* sp.	Zambia	KX833585	KX833404	KX833491	KX833685	Alvarez et al. 2016
*Coniellalimoniformis*	CBS 111021* = PPRI 3870 = CPC 3828	*Fragaria* sp.	South Africa	KX833586	KX833405	KX833492	KX833686	Alvarez et al. 2016
*Coniellalustricola*	DAOMC 251731*	NA	America	MF631778	MF631799	MF651900	MF651899	Raudabaugh et al. 2018
	DAOMC 251732	NA	America	MF631779	MF631800	NA	NA	Raudabaugh et al. 2018
	DAOMC 251733	NA	America	MF631780	MF631801	NA	NA	Raudabaugh et al. 2018
	DAOMC 251734	NA	America	MF631781	MF631802	NA	NA	Raudabaugh et al. 2018
*Coniellamacrospora*	CBS 524.73* = CPC 3935	*Terminalia ivoriensisstem*	Ivory Coast	KX833587	AY339292	KX833493	KX833687	Alvarez et al. 2016
*Coniellamalaysiana*	CBS 141598* = CPC 16659	*Corymbiatorelliana*	Malaysia	KX833588	KX833406	KX833494	KX833688	Alvarez et al. 2016
*Coniellanicotianae*	CBS 875.72* = PD 72/793	*Nicotianatabacum*	Jamaica	KX833590	KX833407	KX833495	KX833690	Alvarez et al. 2016
*Coniellanigra*	CBS 165.60* = IMI 181519 = IMI 181599 = CPC 4198	Soil	India	AY339319	KX833408	KX833496	KX833691	Van Niekerk et al. 2004; Alvarez et al. 2016
*Coniellaobovata*	CBS 111025 = CPC 4196 = IMI 261318	Leaves	South Africa	AY339313	KX833409	KX833497	KX833692	Van Niekerk et al. 2004; Alvarez et al. 2016
*Coniellaparacastaneicola*	CBS 141292* = CPC 20146	*Eucalyptus* sp.	Australia	KX833591	KX833410	KX833498	KX833693	Alvarez et al. 2016
*Coniellaperuensis*	CBS 110394* = RMF 74.01	Soil of rain forest	Peru	KJ710463	KJ710441	KX833499	KX833695	Crous et al. 2015b; Alvarez et al. 2016
*Coniellapseudodiospyri*	CBS 145540* = CPC 35725	*Eucalyptusmicrocorys*	Australia	MK876381	MK876422	MK876479	MK876493	Crous et al. 2019
*Coniellapseudogranati*	CBS 137980* = CPC 22545	*Terminalia stuhlmannii*	Zambia	KJ869132	KJ869189	NA	NA	Crous et al. 2014b
*Coniellapseudokoreana*	MFLU 13-0282* = MFLUCC 12-0427	Leaves	Thailand	MF190145	NA	NA	NA	Senanayake et al. 2017
*Coniellapseudostraminea*	CBS 112624* = IMI 233050	*Fragaria* sp.	South Africa	KX833593	KX833412	KX833500	KX833696	Alvarez et al. 2016
*Coniellaquercicola*	CBS 283.76	Excrements of *Glomerus*, which had eaten forest soil	The Netherlands	KX833594	KX833413	KX833501	KX833697	Alvarez et al. 2016
	CBS 904.69*	*Quercusrobur*	The Netherlands	KX833595	KX833414	KX833502	KX833698	Alvarez et al. 2016
*Coniellasolicola*	CBS 766.71*	Soil	South Africa	KX833597	KX833416	KX833505	KX833701	Alvarez et al. 2016
*Coniellastraminea*	CBS 149.22 = CPC 3932	*Fragaria* sp.	USA	AY339348	AY339296	KX833506	KX833704	Van Niekerk et al. 2004; Alvarez et al. 2016
*Coniellatibouchinae*	CBS 131594* = CPC 18511	*Tibouchinagranulosa*	Brazil	JQ281774	KX833418	KX833507	JQ281778	Miranda et al. 2012; Alvarez et al. 2016
***Coniellaveri***	CGMCC3.27787* = SAUCC 8877-4	***Cinnamomumverum***	**China**	**PQ357098**	**PQ357138**	**PQ361034**	**PQ404810**	**This study**
	**SAUCC 8877-7**	***Cinnamomumverum***	**China**	**PQ357099**	**PQ357139**	**PQ361035**	**PQ404811**	**This study**
*Coniellavitis*	MFLUCC 16-1399* = JZB3700001	*Vitisvinifera*	China	KX890008	KX890083	NA	KX890058	Chethana et al. 2017
*Coniellawangiensis*	CBS 132530* = CPC 19397	*Eucalyptus* sp.	Australia	JX069873	JX069857	KX833509	KX833705	Crous et al. 2012; Alvarez et al. 2016
*Dwiroopalythri*	CBS 109755* = AR 3383	*Lythrumsalicaria*	USA	MN172410	MN172389	MN271801	MN271859	Jiang et al. 2020

**Notes**: New species established in this study are shown in bold. Those marked “*” in the table are represented as ex-type or ex-epitype strains. NA: Not available.

### ﻿Sequence alignment and phylogenetic analyses

The nucleotide sequences of four new species were submitted to the NCBI’s GenBank nucleotide database (https://www.ncbi.nlm.nih.gov/, accessed on 2 Jan. 2025), and all related species were retrieved for phylogenetic analysis. Multiple sequences were aligned using MAFFT version 7 (http://mafft.cbrc.jp/alignment/server/index.html, accessed on 2 Jan. 2025) with default settings, and manual correction was applied if necessary (Katoh et al. 2019). For phylogenetic analyses, single and concatenated sequences were subjected to analysis by Maximum Likelihood (ML) and Bayesian Inference (BI) algorithms, respectively. Both ML and BI were executed on the CIPRES Science Gateway portal (https://www.phylo.org/, accessed on 2 Jan. 2025) or offline software (ML was executed in RaxML-HPC2 on XSEDE v8.2.12 and BI analysis was executed in MrBayes v3.2.7a with 64 threads on Linux) (Miller et al. 2012; Ronquist et al. 2012; Stamatakis 2014). For the ML analysis, the default parameters were used, and 1,000 rapid bootstrap replicates were run with the GTR+G+I model of nucleotide evolution; for BI, it was performed using a rapid bootstrapping algorithm with an automatic stop option and utilized MrModeltest v.2.3 to determine the best evolutionary model for each partition (Nylander 2004; Zhang et al. 2024a). Bayesian Inference posterior probabilities (BIPP) were evaluated by Markov Chain Monte Carlo (MCMC) (Rannala and Yang 1996; Zhaxybayeva and Gogarten 2002). The BI analyses encompassed two parallel runs spanning 5,000,000 generations, with a stop rule incorporated and a sampling frequency of 50 generations. The burn-in fraction was set at 0.25, and posterior probabilities were calculated from the remaining trees. The resulting trees were generated using FigTree v. 1.4.4 (http://tree.bio.ed.ac.uk/software/figtree, accessed on 2 Jan. 2025) or ITOL: Interactive Tree of Life (https://itol.embl.de/, accessed on 2 Jan. 2025) (Letunic and Bork 2021), and the final layout of the trees was refined in Adobe Illustrator CC 2019. The names of the isolates in this study are marked in red in the phylogenetic tree.

## ﻿Results

### ﻿Molecular phylogeny

**Figure 1.**
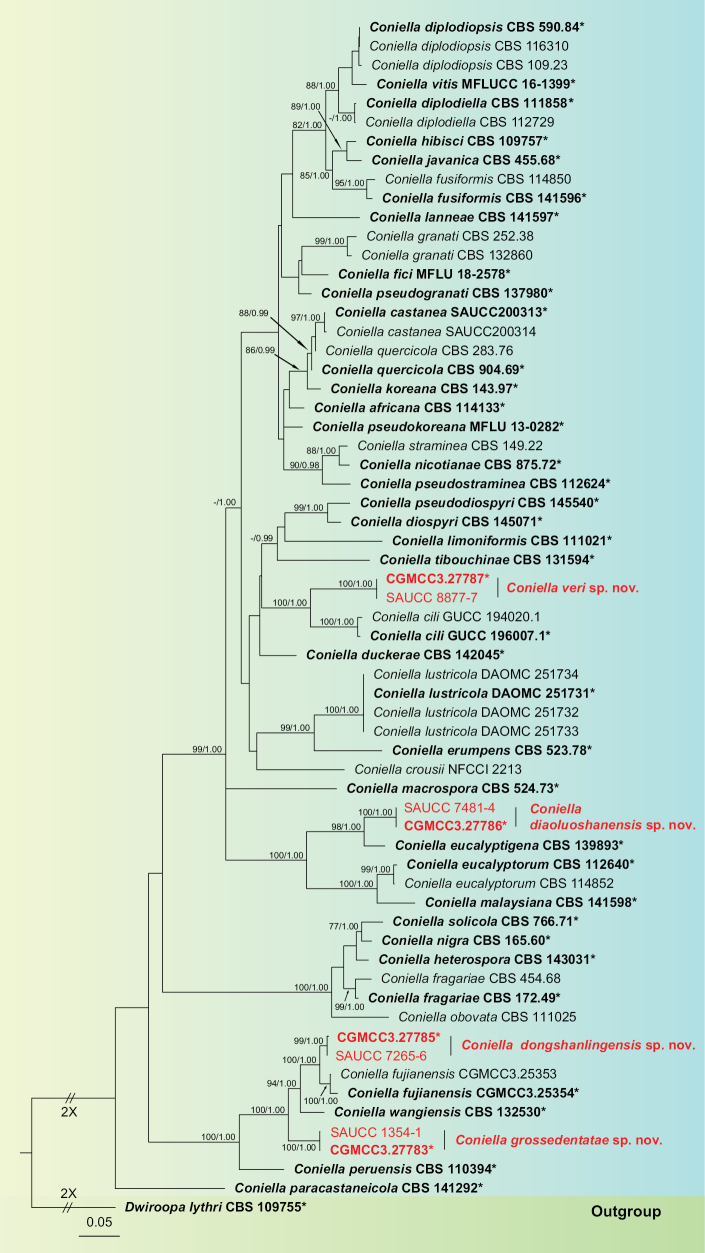
Phylogenetic relationship of *Coniella* based on concatenated sequences of ITS, LSU, *rpb2*, and *tef1-α* sequence data with *Dwiroopalythri* (CBS 109755) as the outgroup. The Maximum Likelihood Bootstrap Value (left, MLBV ≥ 75%) and the Bayesian Inference Posterior Probability (right, BIPP ≥ 0.90) are shown as MLBV/BIPP above the nodes. The ex-type strains are marked with “*” and indicated in boldface. Strains from this study are shown in red. The scale bar at the bottom left represents 0.05 substitutions per site. Some branches are shortened according to the indicated multipliers to fit the page size, and these are indicated by the symbol (//).

Initially, based on the ITS sequence data, we preliminarily determined that the eight strains belong to *Coniella*. Subsequently, based on ML and BI methods, we conducted a combined analysis of ITS, LSU, *rpb2*, and *tef1-α* gene data to construct phylogenetic trees for further determination of the phylogenetic position of these strains. The phylogenetic analysis of *Coniella* strains included 63 sequences, with *Dwiroopalythri* (CBS 109755) serving as the outgroup. The final alignment comprised 2800 concatenated characters, *viz.* 1–600 (ITS), 601–1380 (LSU), 1381–2140 (*rpb2*), and 2141–2800 (*tef1-α*). The ML optimization likelihood was calculated to be -23461.791405. The matrix exhibited 1116 distinct alignment patterns, with 18.42% of characters or gaps remaining undetermined. The optimal models, evaluated by MrModeltest and selected in the BI, are as follows: the SYM+I+G model for ITS and the GTR+I+G model for LSU, *rpb2*, and *tef1-α*. The alignment exhibited a total of 1121 unique site patterns (ITS: 211, LSU: 78, *rpb2*: 322, *tef1-α*: 510). The topology of the ML tree concurred with that derived from BI; thus, only the ML tree is presented (Fig. 1). Combining morphological characteristics and molecular phylogenetic analyses, the eight strains in this study were introduced as four new species, namely *Conielladiaoluoshanensis*, *C.dongshanlingensis*, *C.grossedentatae*, and *C.veri*.

### ﻿Taxonomy

#### ﻿ Coniella diaoluoshanensis

Taxon classificationFungiDiaporthalesSchizoparmaceae

D.H. Li, J.W. Xia & X.G. Zhang sp. nov.

91E8F355-EED2-5FBF-B287-5596963A2E7B

856520

Fig. 2

##### Holotype.

China • Hainan Province: Diaoluoshan National Forest Park, on diseased leaves of *Kadsuralongipedunculata* (Schisandraceae), 18.660546°N, 109.936445°E, 94.1 m asl., 27 Mar. 2024, D.H. Li, holotype HSAUP 7481-1, ex-type living culture SAUCC 7481-1 = CGMCC3.27786.

**Figure 2.**
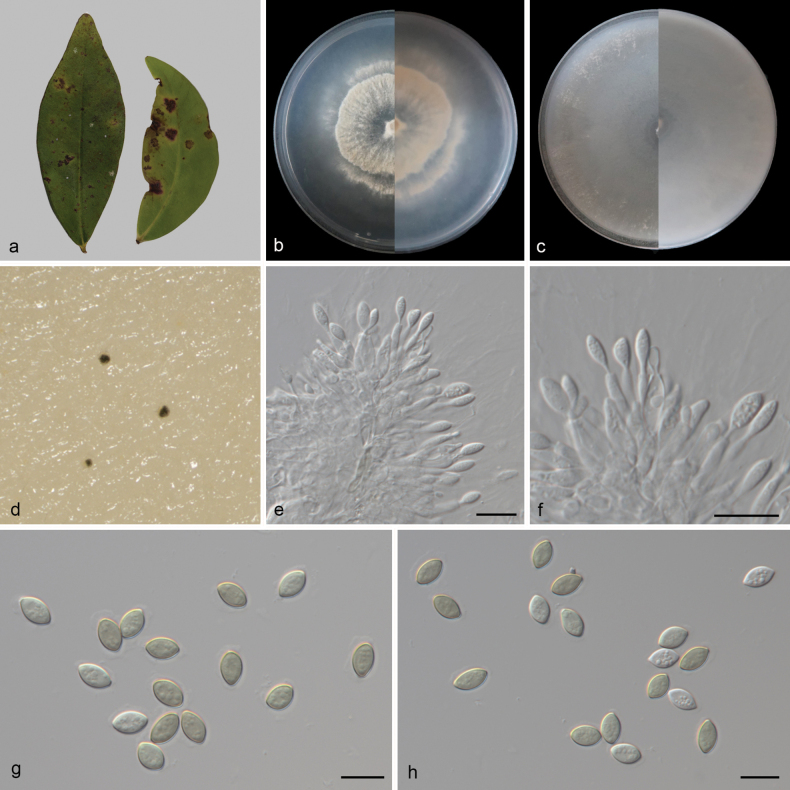
*Conielladiaoluoshanensis* (CGMCC3.27786) **a** leaves of *Kadsuralongipedunculata***b, c** surface and reverse sides of colony after 14 days on PDA (**b**) and OA (**c**) **d** conidiomata forming on OA **e, f** conidiophores and conidiogenous cells with developing conidia **g, h** conidia. Scale bars: 10 μm (**e–h**).

##### Etymology.

Named after the collection site of the type specimen, Diaoluoshan National Forest Park.

##### Description.

***Hypha*** immersed, 1.9–6.5 μm wide, branched, multi-septate, enlarged towards septum and terminal, hyaline. Asexual morph: ***Conidiomata*** nearly spherical, separate, scarce, immersed or superficial, surface uneven, sizes inconsistent, black. ***Conidiophores*** cylindrical, aseptate, straight or slightly curved, densely aggregated, simple, smooth, usually reduced to conidiogenous cells. ***Conidiogenous cells*** phialidic, simple, aggregative, hyaline, smooth, 8.1–11 × 1.4–2.6 μm (mean ± SD = 9.6 ± 0.8 × 2.1 ± 0.4 μm, n = 30), with apical periclinal thickening, blastospore at the apex. ***Conidia*** elliptical or fusiform, apices tapering, subobtuse, apically rounded, widest at the middle, bases tapering to a truncate hilum, multi-guttulate, immature conidia hyaline, mature conidia pale olivaceous, wall darker than pale olivaceous body of conidium, smooth, 7.5–9.3 × 4.7–5.5 μm (mean ± SD = 8.4 ± 0.5 × 5.1 ± 0.3 μm, n = 30). Sexual morph unknown.

##### Culture characteristics.

Colonies on PDA after 14 days of cultivation in the dark at 25 °C, reaching 75–77 mm in diam., with a growth rate of 5.4–5.5 mm/day; from above: white to cream-colored with age, sparse aerial mycelium at the center, irregularly circular, slightly low; peripheral mycelium dense, concentric rings, flat; colony edge irregular, sparse aerial mycelium, dispersed, striped; reverse: similar in color. Colonies on OA covering entire plate after 14 days of cultivation in the dark at 25 °C; from above: white, devoid of aerial mycelium at the center, with dispersed and sparse aerial mycelium at the edges; reverse: even white texture.

##### Additional material studied.

China • Hainan Province: Diaoluoshan National Forest Park, on diseased leaves of *Kadsuralongipedunculata* (Schisandraceae), 18.660546°N, 109.936445°E, 94.1 m asl., 27 Mar. 2024, D.H. Li, HSAUP 7481-4, living culture SAUCC 7481-4.

##### Notes.

Phylogenetic analyses showed that *Conielladiaoluoshanensis* formed an independent clade (Fig. 1) and was closely related to *C.eucalyptigena* (CBS 139893), *C.eucalyptorum* (CBS 112640 and CBS 114852), and *C.malaysiana* (CBS 141598). *Conielladiaoluoshanensis* was distinguished from *C.eucalyptigena* by 4/573 and 7/791 base-pair differences in ITS and LSU sequences, from *C.eucalyptorum* (CBS 112640) by 19/565, 7/793, 68/765, and 164/539 base-pair differences in ITS, LSU, *rpb2*, and *tef1-α* sequences, and from *C.malaysiana* by 16/553, 7/783, 67/767, and 154/488 base-pair differences in ITS, LSU, *rpb2*, and *tef1-α* sequences, respectively. Morphologically, *C.eucalyptigena* lacks asexual sporulation description, making it impossible to compare microscopic structures with *C.diaoluoshanensis*. However, their macroscopic colony colors differ greatly: on PDA, *C.diaoluoshanensis* is cream-colored while *C.eucalyptigena* is salmon; on OA, *C.diaoluoshanensis* is white on the surface, whereas *C.eucalyptigena* is rosy buff. Morphologically, since *C.eucalyptigena* only had a description of sexual morphology, it could not be directly compared with the asexual morphology in this study. Then, *C.eucalyptorum* and *C.malaysiana*, which were closely related on the evolutionary tree, were selected for comparison. The conidiogenous cells of *C.diaoluoshanensis* (8.1–11 × 1.4–2.6 μm) shorter than those of *C.eucalyptorum* (10–17 × 3–3.5 μm) and *C.malaysiana* (8.5–18 × 1.5–3.5 μm); the conidia of *C.diaoluoshanensis* (7.5–9.3 × 4.7–5.5 μm) shorter than those of *C.eucalyptorum* (9–14 × 6–8 μm) and *C.malaysiana* (8–11.5 × 3–5 μm); and the mature conidial color of *C.diaoluoshanensis* (pale olivaceous) was lighter than that of *C.eucalyptorum* (medium to dark red-brown) and *C.malaysiana* (pale brown) (Van Niekerk et al. 2004; Crous et al. 2015a; Alvarez et al. 2016; Zhang et al. 2024b). Therefore, we describe our collection as a novel species.

#### ﻿ Coniella dongshanlingensis

Taxon classificationFungiDiaporthalesSchizoparmaceae

D.H. Li, J.W. Xia & X.G. Zhang sp. nov.

E34003C3-AC81-5F82-B455-84E8DD538AAC

856519

Fig. 3

##### Holotype.

China • Hainan Province: Dongshanling Scenic Area, on diseased leaves of *Lygodiumcircinnatum* (Lygodiaceae), 18.802153°N, 110.421473°E, 18.8 m asl., 26 Mar. 2024, D.H. Li, holotype HSAUP 7265-5, ex-type living culture SAUCC 7265-5 = CGMCC3.27785.

**Figure 3.**
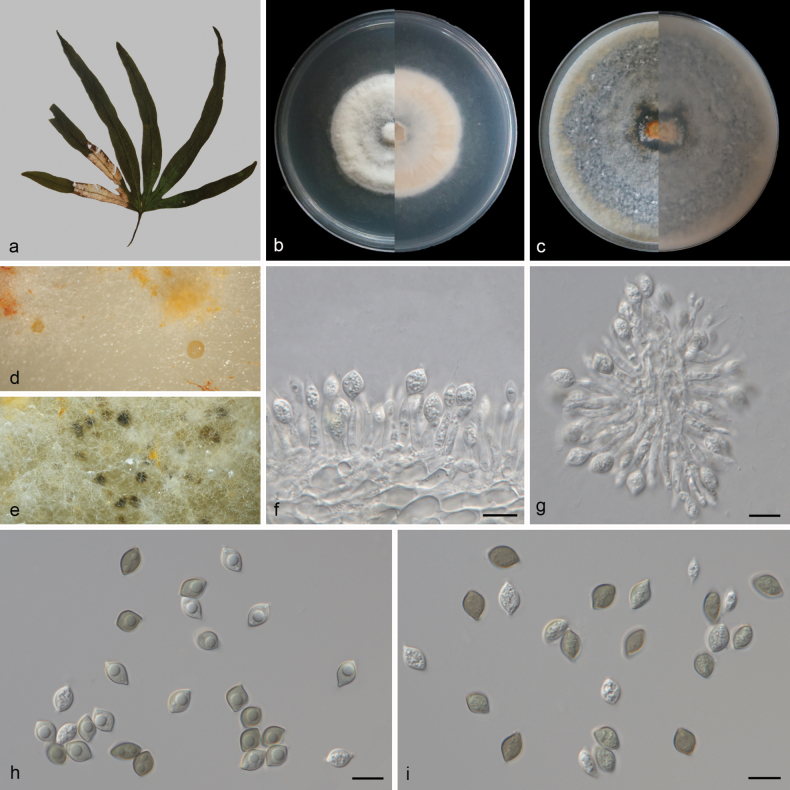
*Conielladongshanlingensis* (CGMCC3.27785) **a** a leaf of *Lygodiumcircinnatum***b, c** surface and reverse sides of colony after 14 days on PDA (**b**) and OA (**c**) **d, e** conidiomata forming on PDA**f, g** conidiophores and conidiogenous cells with developing conidia **h, i** conidia. Scale bars: 10 μm (**f–i**).

##### Etymology.

Named after the collection site of the type specimen, Dongshanling Scenic Area.

##### Description.

***Hypha*** superficial, 1.1–3.2 μm wide, less branched, multi-septate, hyaline to pale yellow. Asexual morph: ***Conidiomata*** pycnidial to nearly spherical, separate, superficial, surface enveloped in a gelatinous sheath, sizes inconsistent, initially appearing hyaline, becoming black with mature. ***Conidiophores*** cylindrical, aseptate, straight or slightly curved, densely aggregated, simple, smooth, usually reduced to conidiogenous cells. ***Conidiogenous cells*** phialidic, simple, aggregative, hyaline, smooth, 7.3–19.2 × 1.5–3.3 μm (mean ± SD = 12.6 ± 2.6 × 2.4 ± 0.5 μm, n = 30), with apical periclinal thickening, blastospore at the apex. ***Conidia*** elliptical to fusiform, apices tapering, subobtuse, apically rounded, bases tapering to a truncate hilum, immature conidia hyaline, multi-guttulate, mature conidia olivaceous, 1–2 guttulate, wall darker than olivaceous body of conidium, smooth, 7.8–10 × 5.1–7 μm (mean ± SD = 8.7 ± 0.6 × 6.2 ± 0.4 μm, n = 30). Sexual morph unknown.

##### Culture characteristics.

Colonies on PDA after 14 days of cultivation in the dark at 25 °C, reaching 47–50 mm in diam., with a growth rate of 3.4–3.6 mm/day; from above: white to pale orange with age, medium aerial mycelium, circular, slightly low at the center, slightly higher at the edges; reverse: similar in color. Colonies on OA covering entire plate after 14 days of cultivation in the dark at 25 °C; from above: pale orange, interspersed with extensive black pycnidia, medium aerial mycelium, flat; reverse: similar in color.

##### Additional material studied.

China • Hainan Province: Dongshanling Scenic Area, on diseased leaves of *Lygodiumcircinnatum* (Lygodiaceae), 18.802153°N, 110.421473°E, 18.8 m asl., 26 Mar. 2024, D.H. Li, HSAUP 7265-6, living culture SAUCC 7265-6.

##### Notes.

Phylogenetic analyses showed that *Conielladongshanlingensis* formed an independent clade (Fig. 1) and was closely related to *C.fujianensis* (CGMCC3.25353 and CGMCC3.25354). *Conielladongshanlingensis* was distinguished from *C.fujianensis* (CGMCC3.25354) by 5/589, 9/657, and 19/306 base-pair differences in ITS, *rpb2*, and *tef1-α* sequences, respectively. Morphologically, the conidiogenous cells of *C.dongshanlingensis* (7.3–19.2 × 1.5–3.3 μm) are longer than those of *C.fujianensis* (3.5–8 × 2.5–3.5 μm); the conidia of *C.dongshanlingensis* (7.8–10 × 5.1–7 μm) slightly shorter than those of *C.fujianensis* (8–10.5 × 5.5–7.5 μm), and the mature conidial color of *C.dongshanlingensis* (olivaceous) is lighter than that of *C.fujianensis* (brown) (Mu et al. 2024). Therefore, we describe our collection as a novel species.

#### ﻿ Coniella grossedentatae

Taxon classificationFungiDiaporthalesSchizoparmaceae

D.H. Li, J.W. Xia & X.G. Zhang sp. nov.

19B869A5-0872-5969-924A-9F0758CE5CF1

856518

Fig. 4

##### Holotype.

China • Fujian Province: Wuyishan City, Xingcun Town, on diseased leaves of *Ampelopsisgrossedentata* (Vitaceae), 27.749556°N, 117.679038°E, 751.68 m asl., 15 Oct. 2022, D.H. Li, holotype HSAUP 1354-3, ex-type living culture SAUCC 1354-3 = CGMCC3.27783.

**Figure 4.**
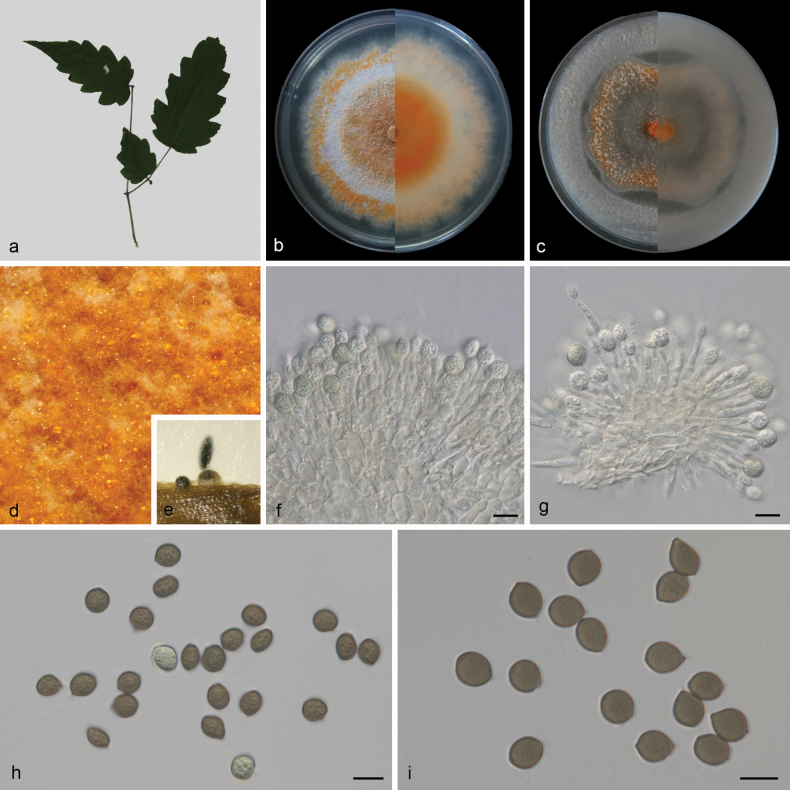
*Coniellagrossedentatae* (CGMCC3.27783) **a** leaves of *Ampelopsisgrossedentata***b, c** surface and reverse sides of colony after 14 days on PDA (**b**) and OA (**c**) **d** colony on PDA**e** conidiomata forming on pine needle **f, g** conidiophores and conidiogenous cells with developing conidia **h, i** conidia. Scale bars: 10 μm **(f–i)**.

##### Etymology.

Named after the species epithet of the host plant, *Ampelopsisgrossedentata*.

##### Description.

***Hypha*** superficial, 1.3–3.5 μm wide, branched, multi-septate, hyaline to pale orange. Asexual morph: ***Conidiomata*** spherical or narrowly ellipsoid, separate, immersed or superficial, some surfaces enveloped in a gelatinous sheath, some surface uneven, sizes inconsistent, black. ***Conidiophores*** cylindrical, aseptate, straight or slightly curved, densely aggregated, simple, usually reduced to conidiogenous cells. ***Conidiogenous cells*** phialidic, simple, aggregative, hyaline, smooth, 10.6–23.1 × 1.7–3.8 μm (mean ± SD = 16.8 ± 3 × 2.5 ± 0.6 μm, n = 30), with apical periclinal thickening, blastospore at the apex. ***Conidia*** nearly spherical, apices acute, widest at the middle, bases tapering to a truncate hilum, multi-guttulate, immature conidia hyaline, mature conidia medium brown, wall darker than medium brown body of conidium, smooth, 8–10.5 × 7.5–9.5 μm (mean ± SD = 9.4 ± 0.6 × 8.4 ± 0.5 μm, n = 30). Sexual morph unknown.

##### Culture characteristics.

Colonies on PDA after 14 days of cultivation in the dark at 25 °C, reaching 86–90 mm in diam., with a growth rate of 6.1–6.4 mm/day; from above: orange in the middle and edges, with white in between, medium aerial mycelium, granular, circular, flat; reverse: similar in color. Colonies on OA covering entire plate after 14 days of cultivation in the dark at 25 °C; from above: white in the middle and edges, with orange in between, sparse aerial mycelium, flat; reverse: similar in color.

##### Additional material studied.

China • Fujian Province: Wuyishan City, Xingcun Town, on diseased leaves of *Ampelopsisgrossedentata* (Vitaceae), 27.749556°N, 117.679038°E, 751.68 m asl., 15 Oct. 2022, D.H. Li, HSAUP 1354-1, living culture SAUCC 1354-1.

##### Notes.

Phylogenetic analyses showed that *Coniellagrossedentatae* formed an independent clade (Fig. 1) basal to *C.dongshanlingensis* (CGMCC3.27785, SAUCC 7265-6), *C.fujianensis* (CGMCC 3.25353, CGMCC 3.25354), and *C.wangiensis* (CBS 132530). *Coniellagrossedentatae* can be distinguished from *C.dongshanlingensis* by 4/604, 1/793, 52/902, and 80/532 base-pair differences in ITS, LSU, *rpb2*, and *tef1-α* sequences, and from *C.fujianensis* by 8/588, 1/798, 34/657, and 64/313 base-pair differences in ITS, LSU, *rpb2*, and *tef1-α* sequences, and from *C.wangiensis* by 2/603, 5/798, 35/767, and 79/329 base-pair differences in ITS, LSU, *rpb2*, and *tef1-α* sequences, respectively. Morphologically, the conidiogenous cells of *C.grossedentatae* (10.6–23.1 × 1.7–3.8 μm) are longer than those of *C.dongshanlingensis* (7.3–19.2 × 1.5–3.3 μm), *C.fujianensis* (3.5–8 × 2.5–3.5 μm), and *C.wangiensis* (15–20 × 3–4 μm); the conidia of *C.grossedentatae* (8–10.5 × 7.5–9.5 μm) are wider than those of *C.dongshanlingensis* (7.8–10 × 5.1–7 μm) and *C.fujianensis* (8–10.5 × 5.5–7.5 μm), and shorter than those of *C.wangiensis* (9–13 × 7–10 μm) (Crous et al. 2012; Alvarez et al. 2016). Therefore, we describe our collection as a novel species.

#### ﻿ Coniella veri

Taxon classificationFungiDiaporthalesSchizoparmaceae

D.H. Li, J.W. Xia & X.G. Zhang sp. nov.

F81D79DD-4D9E-5E42-B7E7-6B3E0082EE92

856521

Fig. 5

##### Holotype.

China • Yunnan Province: Pu’er City, Yixiang Town, Pu’er Sun River Forest Park, on diseased leaves of *Cinnamomumverum* (Lauraceae), 22.593953°N, 101.086217°E, 1596.44 m asl., 15 May 2024, D.H. Li, holotype HSAUP 8877-4, ex-type living culture SAUCC 8877-4 = CGMCC3.27787.

**Figure 5.**
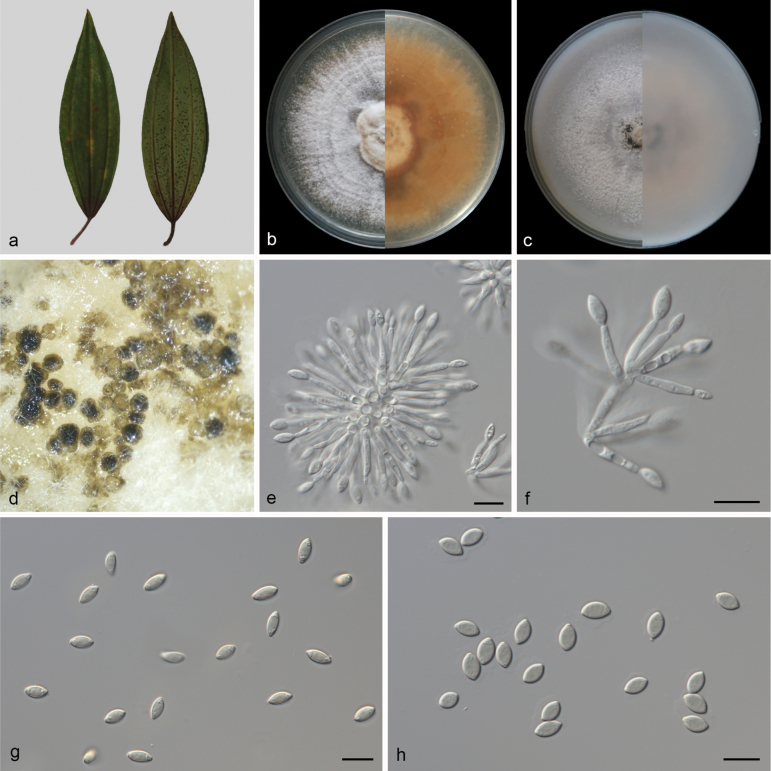
*Coniellaveri* (CGMCC3.27787) **a** leaves of *Cinnamomumverum***b, c** surface and reverse sides of colony after 14 days on PDA (**b**) and OA (**c**) **d** conidiomata forming on OA **e, f** conidiophores and conidiogenous cells with developing conidia **g, h** conidia. Scale bars: 10 μm (**e–h**).

##### Etymology.

Named after the species epithet of the host plant, *Cinnamomumverum*.

##### Description.

***Hypha*** superficial, 1.3–3.3 μm wide, branched, multi-septate, hyaline. Asexual morph: ***Conidiomata*** spherical, aggregated or solitary, immersed or superficial, some surfaces enveloped in a gelatinous sheath, some surface uneven, sizes inconsistent, initially appearing hyaline, becoming black with mature. ***Conidiophores*** cylindrical, septate, branched, straight or slightly curved, densely aggregated, simple, usually reduced to conidiogenous cells. ***Conidiogenous cells*** phialidic, simple, aggregative, or solitary, hyaline, smooth, 9.5–17.5 × 1.2–2.5 μm (mean ± SD = 12.5 ± 1.5 × 1.8 ± 0.4 μm, n = 30), with apical periclinal thickening, blastospore at the apex. ***Conidia*** elliptical to fusiform, apices acute, widest at the middle, bases tapering to a truncate hilum, multi-guttulate gather at both ends, hyaline, thick-walled, smooth, 6.2–8.8 × 3.6–4.7 μm (mean ± SD = 7.7 ± 0.6 × 4 ± 0.3 μm, n = 30). Sexual morph unknown.

##### Culture characteristics.

Colonies on PDA after 14 days of cultivation in the dark at 25 °C, reaching 81–85 mm in diam., with a growth rate of 5.8–6.1 mm/day; from above: white, medium aerial mycelium, slightly higher at the center, circular, radial, flat; reverse: pale orange in the middle, orange in the edges. Colonies on OA after 14 days of cultivation in the dark at 25 °C, reaching 72–77 mm in diam., had a growth rate of 5.1–5.5 mm/day; from above: white, sparse aerial mycelium, black pycnidia formed in the center, flat; reverse: similar in color.

##### Additional material studied.

China • Yunnan Province: Pu’er City, Yixiang Town, Pu’er Sun River Forest Park, on diseased leaves of *Cinnamomumverum* (Lauraceae), 22.593953°N, 101.086217°E, 1596.44 m asl., 15 May 2024, D.H. Li, HSAUP 8877-7, living culture SAUCC 8877-7.

##### Notes.

Phylogenetic analyses showed that *Coniellaveri* formed an independent clade (Fig. 1) and was closely related to *C.cili* (GUCC 194020.1 and GUCC 196007.1). *Coniellaveri* can be distinguished from *C.cili* (GUCC 196007.1) by 31/597, 8/791, 52/869, and 125/516 base-pair differences in ITS, LSU, *rpb2*, and *tef1-α* sequences, respectively. Morphologically, the conidiogenous cells of *C.veri* (9.5–17.5 × 1.2–2.5 μm) are shorter than those of *C.cili* (13–23.5 × 1–2 μm); the conidia of *C.veri* (6.2–8.8 × 3.6–4.7 μm) are shorter than those of *C.cili* (5.5–17.5 × 2.5–5 μm); the conidial shape of *C.veri* is elliptical to fusiform, whereas the conidial size and shape of *C.cili* exhibit considerable variation, including limoniform, fusoid, clavate, cylindrical, and elongated elliptical forms (Zhang et al. 2024b). Therefore, we describe our collection as a novel species.

## ﻿Discussion

*Coniella* species have a worldwide distribution, reported in countries across all continents (Van Niekerk et al. 2004; Alvarez et al. 2016). They have been found in Asia (e.g., China, India, Indonesia, Malaysia, South Korea, and Thailand), Europe (e.g., Belgium, Denmark, France, Italy, the Netherlands, Switzerland, and Spain), Africa (e.g., Ivory Coast, South Africa, and Zambia), the Americas (e.g., the United States, Brazil, Peru, Jamaica, and Chile), and Oceania (e.g., Australia). These countries, ranging from landlocked nations such as Zambia and Switzerland to coastal countries like China, Brazil, and Australia, as well as island nations including Jamaica and Indonesia, are geographically diverse. They are distributed on both sides of the equator and span multiple climatic zones, from tropical to frigid, coastal to inland, and plain to mountain, encompassing diverse climate types such as tropical, temperate, and alpine. Many countries, including most of Africa, northern Brazil, Indonesia, and Malaysia, have tropical climates with high temperatures and abundant precipitation year-round. China, with its vast territory, large latitudinal span, wide longitudinal extent, and complex and diverse topography, nearly covers all major climate types, providing favorable conditions for the formation of *Coniella* species diversity (Castlebury et al. 2002; Van Niekerk et al. 2004; Alvarez et al. 2016; Raudabaugh et al. 2018; Wang et al. 2022; Mu et al. 2024; Zhang et al. 2024b).

Currently, *Coniella* has accepted 66 species, many of which were introduced solely based on morphological studies (Index Fungorum: https://indexfungorum.org; MycoBank: http://www.mycobank.org; Alvarez et al. 2016; Mu et al. 2024). Morphological characteristics of some conidia are highly similar and can be classified into two categories: one comprises olivaceous brown to brown conidia that are ellipsoid or globose, while the other category consists of hyaline conidia that are fusiform or clavate, often with very similar shapes and sizes. Rendering precise identification of *Coniella* species difficult solely on morphological characteristics (Crous et al. 2014a). Consequently, there is a strong current trend towards integrating morphological and molecular methods to assess or clarify the taxonomic placement and phylogenetic relationships of *Coniella* species (Alvarez et al. 2016). Based on phylogenetic analyses of ITS, LSU, and *tef1-α* sequence data, Van Niekerk et al. (2004) demonstrated that *Coniella* represents a distinct evolutionary lineage within the Diaporthales (Van Niekerk et al. 2004). Based on phylogenetic analyses of ITS, LSU, *rpb2*, and *tef1-α* sequence data, Alvarez et al. (2016) conducted a taxonomic revision of the genus. Since then, phylogenetic analyses of *Coniella* have largely continued to use these four genetic loci (Alvarez et al. 2016).

According to previous studies, *Coniella* species have been recorded as plant pathogens, endophytes, and saprobes (Samuels et al. 1993; Ferreira et al. 1997; Alvarez et al. 2016; Chethana et al. 2017). Their hosts encompass multiple categories, including plants (such as trees, shrubs, herbs, and ferns), animal excreta, and soils (Crous et al. 2015b; Alvarez et al. 2016). In recent years, several *Coniella* species have been reported and described in China. For example, Fröhlich and Hyde (2000) discovered *C.calamicola* on both living and dead leaves of *Daemonoropsmargaritae* in Hong Kong. Chen et al. (2014) first reported that *C.granati* can cause fruit rot and twig blight in pomegranate (*Punicagranatum*) in Anhui Province. Chethana et al. (2017) reported that *C.vitis* is the pathogenic fungus causing white rot in grapes (*Vitisvinifera*) in Beijing Municipality, Guangxi, Hebei, Henan, and Jilin Provinces. Tennakoon et al. (2021) isolated a new species, *C.fici*, from dead leaves of *Ficusseptica* (Moraceae) on the island of Taiwan. Wang et al. (2022) isolated a new species, *C.castanea*, from symptomatic leaves of *Castaneamollissima* (Fagaceae) in an orchard in Shandong Province. Mu et al. (2024) isolated a new species, *C.fujianensis*, from diseased leaves of *Canariumalbum* (Burseraceae) in Fujian Province. Zhang et al. (2024b) isolated the endophytic species *C.cili* from healthy fruits and seeds of *Rosaroxburghii* (Rosaceae) in Guizhou Province.

During a continuous survey of terrestrial plant fungi in certain regions of southern China, four new species of *Coniella* were discovered from diseased leaf tissues of infected plants in Fujian, Hainan, and Yunnan provinces. These new species are named *Conielladiaoluoshanensis*, *C.dongshanlingensis*, *C.grossedentatae*, and *C.veri*. Among them, *C.grossedentatae* utilizes *Ampelopsisgrossedentata* (Vitaceae) as its host. Van Niekerk et al. (2004) have previously reported species of *C.diplodiopsis* isolated from *Vitisvinifera* (Vitaceae) collected in Italy. In contrast, *C.diaoluoshanensis*, *C.dongshanlingensis*, and *C.veri* are the first reports that are associated with the hosts *Kadsuralongipedunculata*, *Lygodiumcircinnatum*, and *Cinnamomumverum*, respectively. This will further broaden the host range of *Coniella* species and contribute to the fields of plant pathology and fungal taxonomy. With the increasing number of *Coniella* species, we believe that comprehensive research on this genus will uncover more hidden *Coniella* species from terrestrial plants.

## Supplementary Material

XML Treatment for Coniella diaoluoshanensis

XML Treatment for Coniella dongshanlingensis

XML Treatment for Coniella grossedentatae

XML Treatment for Coniella veri

## ﻿Additional information

### Author ORCIDs

Duhua Li https://orcid.org/0009-0006-5200-2034

Qiyun Liu https://orcid.org/0009-0009-9545-7962

Zhaoxue Zhang https://orcid.org/0000-0002-4824-9716

Xiuguo Zhang https://orcid.org/0000-0001-9733-8494

Jiwen Xia https://orcid.org/0000-0002-7436-7249

### Data availability

All of the data that support the findings of this study are available in the main text.
